# 人血清中97种典型化学污染物暴露特征及健康风险评估

**DOI:** 10.3724/SP.J.1123.2023.11022

**Published:** 2024-02-08

**Authors:** Mingye ZHANG, Yan CAO, Xiang LI, Jing KOU, Qitong XU, Sijie YANG, Zhiyi ZHENG, Jun LIU, Surong MEI

**Affiliations:** 华中科技大学公共卫生学院环境医学研究所, 教育部环境与健康重点实验室, 湖北 武汉 430030; Key Laboratory of Environment & Health of Ministry of Education, Institute of Environmental Medicine, School of Public Health, Huazhong University of Science and Technology, Wuhan 430030, China

**Keywords:** 固相萃取, 气相色谱-串联质谱, 化学污染物, 血清, 健康风险评估, solid-phase extraction (SPE), gas chromatography-tandem mass spectrometry (GC-MS/MS), chemical pollutants, serum, health risk assessment

## Abstract

本研究以我国中部地区某市60名普通成人为研究对象,采用固相萃取结合气相色谱-串联质谱技术(SPE-GC-MS/MS)同时测定人血清样品中97种典型化学污染物的浓度,采用多元线性回归模型分析人口学特征、生活习惯和饮食对血清中化学污染物浓度的影响,结合已知的生物监测当量(BEs)和人类生物监测(HBM)值计算危害商(HQ)和危害指数(HI)来评估化学污染物单一和累积暴露的健康风险。结果表明,在人体血清中主要检出的污染物为有机氯农药(OCPs)、多氯联苯(PCBs)及多环芳烃(PAHs),其中六氯苯(100.0%)、五氯苯酚(100.0%)、*p*,*p*'-滴滴伊(100.0%)、PCB-138(100.0%)、PCB-153(98.3%)、*β*-六六六(91.7%)、芴(85.0%)和蒽(75.0%)的检出率大于70%。女性血清中*β*-六六六的浓度水平高于男性;年龄与*p*,*p*'-滴滴伊、PCB-138、PCB-153和*β*-六六六暴露水平呈正相关;*p*,*p*'-滴滴伊和*β*-六六六暴露水平增高可能与肉类摄入频率高有关,蔬菜摄入频率高的人群具有较高的血清五氯苯酚暴露水平。6.7%的成人血清中五氯苯酚HQ大于1,在研究人群中未观察到六氯苯和p,p-滴滴伊暴露的风险,28.3%的研究对象HI值大于1。总体而言,该地区普通成人广泛暴露于各种化学污染物,性别、年龄和饮食可能是影响化学污染物浓度的主要因素,且化学污染物单一和复合暴露的健康风险不容忽视。

## Abstract (en)

Rapid industrial and agricultural developments in China have led to the wide use and discharge of chemical products and pesticides, resulting in extensive residues in environmental media. These residues can enter the human body through various pathways, leading to high exposure risks and health hazards. Because the human body is exposed to a variety of chemical pollutants, accurately quantifying the exposure levels of these pollutants in the human body and evaluating their health risks are of great importance. In this study, the serum concentrations of 97 typical chemical pollutants of 60 adults in central China were simultaneously determined using solid-phase extraction coupled with gas chromatography-tandem mass spectrometry (SPE-GC-MS/MS). In this method, 200 μL of a serum sample was mixed with 10 μL of an isotope-labeled internal standard solution. The sample was vortexed and refrigerated overnight at 4 ℃. Each sample was then deproteinized by the addition of 200 μL of 15% formic acid aqueous solution and vortexed. The serum sample was loaded into a preconditioned Oasis^®^ PRiME HLB SPE cartridge and rinsed with 3 mL of methanol-water (6∶1, v/v). The SPE cartridge was subsequently vacuumed. The analytes were eluted with 3 mL of dichloromethane followed by 3 mL of n-hexane. The eluent was concentrated to near dryness under a gentle nitrogen stream and reconstituted with 100 μL of acetone. The samples were determined by GC-MS/MS and separated on a DB-5MS capillary column (30 m×0.25 mm×0.25 μm) with temperature programming. The column temperature was maintained at 70 ℃ for 2 min, increased at a rate of 25 ℃/min to 150 ℃, increased at a rate of 3 ℃/min to 200 ℃, and then held for 2 min. Finally, the column temperature was increased at a rate of 8 ℃/min to 300 ℃ and maintained at this temperature for 8 min. The samples were detected in multiple-reaction monitoring (MRM) mode and quantitatively analyzed using the internal standard method. Multiple linear regression models were used to analyze the effects of demographic characteristics, lifestyle habits, and diet on the concentrations of the chemical pollutants in the serum samples, and known biomonitoring equivalents (BEs) and human biomonitoring (HBM) values were combined to compute hazard quotients (HQs) and hazard indices (HIs) and evaluate the health risks of single and cumulative exposures to the chemical pollutants. The results showed that the main pollutants detected in human serum were organochlorine pesticides (OCPs), polychlorinated biphenyls (PCBs), and polycyclic aromatic hydrocarbons (PAHs). The detection rates of eight pollutants, including hexachlorobenzene (HCB) (100%), pentachlorophenol (PCP) (100%), *p*,*p*'-dichlorodiphenylene (*p*,*p*'-DDE) (100%), PCB-138 (100%), PCB-153 (98.3%), *β*-hexachlorocyclohexane (*β*-HCH) (91.7%), fluorene (Flu) (85.0%), and anthracene (Ant) (75.0%), were greater than 70%. The serum levels of *β*-HCH were higher in females than in males, and age was positively correlated with exposure to *p*,*p*'-DDE, PCB-138, PCB-153, and *β*-HCH. Increased exposure levels to *p*,*p*'-DDE and *β*-HCH may be associated with a high frequency of meat intake, whereas increased exposure level to PCP may be associated with a high frequency of vegetable intake. The serum HQ of PCP was greater than 1 in 6.7% of the samples, and no risk was observed for HCB and *p*,*p*'-DDE exposure in the study population. Approximately 28.3% of the study subjects had HI values greater than 1. Overall, the general adult population in this region is widely exposed to a wide range of chemical pollutants, and gender, age, and diet are likely to be the main factors influencing the concentration of chemical pollutants. The health risk of single and compound exposures to chemical pollutants should not be ignored.

化学污染物是指会对环境造成污染的化学物质,典型的化学污染物主要包括有机氯农药(organochlorine pesticides, OCPs)、多环芳烃(polycyclic aromatic hydrocarbons, PAHs)、多氯联苯(polychlorinated biphenyls, PCBs)、多溴二苯醚(polybrominated diphenyl ethers, PBDEs)等。这些物质可作为杀虫剂、除草剂、杀菌剂、增塑剂和阻燃剂等被广泛应用于农业和工业生产中^[1]^。农药施用和工业化学品排放导致环境介质中化学污染物的广泛残留^[2]^,并通过饮食、空气吸入、皮肤接触等多种途径进入人体,随血液循环到达全身各个组织器官,引起不良健康效应,如内分泌干扰毒性、肝毒性、生殖毒性和免疫毒性等^[3,4]^。

美国^[5]^、加拿大^[6]^和德国^[7]^等发达国家已在全国范围内开展化学污染物内暴露评估,有助于准确评估化学污染物对人体的健康风险。中国疾病预防控制中心也启动了中国人体生物监测项目^[8]^,旨在评估我国人群体内各种化学污染物的残留水平。这些生物监测项目中一种检测方法通常只针对某一类或少数几类物质进行同时检测,需要大量的生物样本、检测时间长且成本高。鉴于人体处于化学污染物复合暴露状态,同时检测多种污染物的内暴露水平具有重要意义。固相萃取技术(SPE)可以对样品同时进行富集和净化且操作简单,常用于样品前处理^[9]^,色谱-串联质谱(MS/MS)技术具有灵敏度高和特异性强等特点,常用于化学污染物的定性定量分析^[10]^。本研究主要关注的化学污染物为沸点低且热稳定性较好的有机化合物,故适合用气相色谱(GC)-MS/MS来进行分析。

血清中化学污染物水平受多种因素的影响,包括人口学特征、生活方式和饮食等,但目前研究纳入的化学污染物种类有限且结论不完全一致^[11,12]^,因此需要进一步研究探讨血清化学污染物浓度水平的影响因素。

基于此,本研究选取我国中部地区普通人群作为研究对象,采用SPE-GC-MS/MS技术同时测定血清中97种化学污染物的内暴露水平,阐明化学污染物暴露水平相关的影响因素,并评估污染物单一和累积暴露的健康风险。

## 1 实验部分

### 1.1 仪器、试剂与材料

8890GC气相色谱仪、7000D-MS三重四极杆质谱仪及真空固相萃取装置(美国Agilent公司);隔膜真空泵(美国GAST公司),氮气发生器(武汉科林普丰仪器有限公司),氮吹仪(北京赛多利斯科学仪器有限公司),涡旋振荡仪(美国Scilogex公司)。

Oasis^®^ PRiME HLB固相萃取柱(1 mL/30 mg,美国Waters公司),二氯甲烷、丙酮(色谱纯,美国Fisher公司),甲醇、正己烷(色谱纯,德国Merck公司), 88%甲酸(分析纯,国药集团有限公司),纯净水(杭州娃哈哈集团有限公司)。

标准品:30种杀虫剂混合标准溶液(28种OCPs和2种氨基甲酸酯类杀虫剂,100 μg/mL)、22种杀虫剂混合标准溶液(13种有机磷杀虫剂和9种拟除虫菊酯类杀虫剂,100 μg/mL)、12种PAHs混合标准溶液(100 μg/mL)购自天津阿尔塔科技有限公司;18种PCBs混合标准溶液(100 μg/mL)、PCB-183标准溶液(100 μg/mL)购自美国AccuStandard公司;7种PBDEs混合标准溶液(50 μg/mL)、腐霉利(100 μg/mL)、乙烯菌核利(100 μg/mL)、虫螨腈(100 μg/mL)和炔螨特(100 μg/mL)标准溶液购自上海安谱实验科技股份有限公司;氟乐灵标准品(0.25 g)、乙菌利标准溶液(100 μg/mL)和异菌脲标准品(0.1 g)购自德国Dr. Ehrenstorfer公司。

同位素内标:二嗪农-D_10_(2.5 mg)、毒死蜱-D_10_(1 mg)、醚菊酯-D_5_(20 mg)购自上海甄准生物科技有限公司;^13^C_12_-*p*,*p*'-DDE(100 μg/mL)、^13^C_12_-*p*,*p*'-DDD(100 μg/mL)、^13^C_12_-*p*,*p*'-DDT(100 μg/mL)购自美国Cambridge Isotope Laboratories公司;芘-D_10_(100 μg/mL)购自加拿大Toronto Research Chemicals公司,PCB-156-D_3_(100 μg/mL)购自天津阿尔塔科技有限公司。

### 1.2 标准溶液配制

将上述标准品溶于丙酮,配制成质量浓度为20 μg/mL的97种化学污染物混合标准贮备液;将混合标准贮备液用丙酮稀释得到质量浓度为2 μg/mL的混合标准应用溶液,并用丙酮配制成质量浓度为0.2、0.5、1、2、5、10、20、50、100、200、500、1000 ng/mL的系列标准溶液。称(量)取一定量内标溶于丙酮,配制成质量浓度为20 ng/mL的内标混合贮备液,于-20 ℃冰箱保存备用。

### 1.3 血液样品采集

本研究从我国中部地区某三甲医院健康管理中心随机招募体检人群作为研究对象,进行问卷调查,收集了60名成人的性别、年龄、教育程度、身高、体重、体质量指数(BMI)、吸烟、饮酒、体育锻炼和膳食资料(肉类、蛋类、奶制品、蔬菜、水果和坚果)等信息。采集研究对象的血液样本,离心取上清液后转入-80 ℃冰箱保存。本研究得到华中科技大学同济医学院伦理委员会批准(No. 2018-IEC-S329),招募对象均签署知情同意书。

### 1.4 样品前处理

取200 μL血清样品于离心管中,加入10 μL质量浓度为20 ng/mL的内标混合贮备液,涡旋混匀后置于4 ℃冰箱中过夜;向血清样品中加入200 μL 15%甲酸水溶液并混匀;样品用Oasis^®^ PRiME HLB固相萃取柱净化,上样前用3 mL二氯甲烷、3 mL甲醇和3 mL纯净水预先活化小柱;上样后分别用1 mL甲醇-水(体积比为1∶6)溶液润洗离心管两次并淋洗小柱,真空抽干;依次用3 mL二氯甲烷和3 mL正己烷进行洗脱,收集全部洗脱液并用氮气吹干,使用100 μL丙酮进行复溶,转移至进样瓶待检测。

### 1.5 分析条件

#### 1.5.1 色谱条件

色谱柱:Agilent DB-5MS毛细管柱(30 m×0.25 mm×0.25 μm);载气流速1.2 mL/min;进样口温度:270 ℃;程序升温:起始温度70 ℃,维持2 min, 25 ℃/min升温至150 ℃;以3 ℃/min升温至200 ℃,维持2 min;以8 ℃/min升温至300 ℃,维持8 min。不分流进样,进样量1 μL。

#### 1.5.2 质谱条件

电子轰击离子源(EI),电离电压70 eV;离子源温度300 ℃,传输线温度300 ℃;溶剂延迟时间6 min;扫描模式为多反应监测(MRM),内标法定量。97种目标物的质谱分析参数详见前期工作^[13]^。

### 1.6 脂质标准化浓度的计算

血清中总胆固醇(total cholesterol, TC)和甘油三酯(triglycerides, TG)含量使用全自动生化分析仪(Cobas 8000,罗氏公司)进行测定,污染物的脂质标准化浓度(ng/g脂质)由式(1)和式(2)^[14]^进行计算。


(1) Total lipids (g/L)=1.12×TC(g/L)+1.33×TG(g/L)+1.48(g/L)

(2) $$C_{\text{Lipid-standardized }}\mathrm{(ng/g lipid)=}\frac{C_{Wet-weighted}(ng/mL)\times 1000}{Total\;lipids(g/L)}$$

其中Total lipids为血清总脂质,*C*_Wet-weight_为污染物的检测浓度,*C*_Lipid-standardized_为污染物的脂质标准化浓度。

### 1.7 统计分析

本研究对检出率大于70%的化学污染物进行统计分析,低于检出限(LOD)的浓度以LOD/ $\sqrt{2}$ 计算。由于污染物的浓度呈偏态分布,因此将其经自然对数转换后纳入多元线性回归模型进行分析。采用R 4.3.1进行统计分析,双侧检验水准*α*=0.05。

### 1.8 健康风险评估

危害商(hazard quotient, HQ)是健康风险评估的重要指标,通过化学污染物实际暴露量与生物监测当量(biomonitoring equivalents, BEs)或人类生物监测(human biomonitoring, HBM)值之比来反映暴露风险,计算公式见式(3)。


(3) $$\mathrm{HQ=}\frac{C_{serum}}{BE/HBM}$$

其中*C*_serum_为血清中化学污染物的浓度;BE为生物介质中污染物或其代谢物的浓度或浓度范围,与暴露指导值(参考剂量、最低风险水平和每日耐受摄入量)一致^[15]^。HBM值是由德国HBM委员会制定的,包括HBM-Ⅰ和HBM-Ⅱ值^[16]^。HBM-Ⅰ值是一个控制值,低于该值预计不会对健康产生不利影响,HBM-Ⅱ值是一个行动水平,超过该值可能会发生相关的不良健康影响,当浓度高于HBM-Ⅰ而低于HBM-Ⅱ值时,无法完全排除对健康的潜在损害。HQ<1时,认为化学污染物暴露无明显的健康风险;HQ>1时,认为化学污染物暴露存在健康风险。危害指数(hazard index, HI)用来估计化学污染物的复合暴露风险^[17]^,计算公式见式(4)。


(4) HI=∑HQ

HI<1时,认为化学污染物复合暴露无明显的健康风险;HI>1时,认为化学污染物复合暴露存在健康风险。

## 2 结果与讨论

### 2.1 人口学特征

本研究纳入的研究对象中女性人数为30名(50.0%),平均年龄为44.62±9.02岁,BMI为(24.16±2.82) kg/m^2^; 28名(46.7%)研究对象教育程度为大学及以上,超过半数的人不吸烟(73.3%)和不饮酒(76.7%)。

### 2.2 血清中化学污染物浓度水平

本研究共检测了血清中97种化学污染物(名单见附表S1,www.chrom-China.com),由浓度水平测定结果可以看出,血清中OCPs、PCBs和PAHs的检出率较高,且以OCPs为主;检出率高于70%的物质有8种,从高到低依次为六氯苯(100.0%)、五氯苯酚(100.0%)、*p*,*p*'-滴滴伊(100.0%)、PCB-138(100.0%)、PCB-153(98.3%)、*β*-六六六(91.7%)、芴(85.0%)和蒽(75.0%),中位数浓度依次为0.600、7.340、2.938、0.013、0.013、0.084、2.020、0.138 ng/mL(见表1)。本研究中六氯苯和五氯苯酚的中位数浓度水平高于德国博物馆职工^[18]^和比利时居民^[19]^等发达国家人群暴露水平;*p*,*p*'-滴滴伊和*β*-六六六的中位数浓度高于黎巴嫩成人^[20]^和韩国普通人群暴露水平^[21]^;这可能是因为我国作为农业大国,农药使用量多^[22,23]^,大规模的生产和使用导致我国受到较为严重的污染。PCB-138和PCB-153的中位数浓度低于美国成年女性^[24]^和加拿大普通人群^[25]^暴露水平;这可能是因为我国不是PCBs主要生产地,总产量仅占全球产量的0.7%^[26]^,污染程度相对较轻。芴和蒽的中位数浓度高于美国军人^[27]^体内浓度水平,这可能是因为PAHs主要来源于化学工业污染、交通尾气污染等,我国PAHs排放较多^[28]^,污染程度不容忽视。总之,血清中污染物浓度水平存在地区差异,这可能与不同地区的经济水平、工业生产、交通状况和人群的生活方式及饮食习惯等有关。

**表1:** 普通人群体内化学污染物浓度分布

Compound		Detection rate/%		Mass concentration in wet-weight/(ng/mL)					*C*_Lipid-standardized_ /(ng/g lipid)				
				Geometric mean	Percentile				Geometric mean	Percentile			
					P25	Median	P75			P25	Median	P75	
HCB	100		0.539		0.325	0.600	0.861		92.279	59.361	103.733		154.898
PCP	100		5.838		2.224	7.340	13.717		999.414	432.995	1173.031		2723.836
*p*,*p*'-DDE	100		2.860		1.412	2.938	5.678		489.556	225.669	434.202		1012.770
PCB-138	100		0.013		0.009	0.013	0.020		2.226	1.513	2.148		3.080
PCB-153	98.3		0.012		0.007	0.013	0.020		2.092	1.379	2.067		3.136
*β*-HCH	91.7		0.080		0.041	0.084	0.209		13.704	7.319	14.060		35.174
Flu	85.0		0.683		0.290	2.020	3.142		116.902	56.988	325.657		531.834
Ant	75.0		0.143		0.050	0.138	0.300		24.442	8.068	23.197		56.790

HCB: hexachlorobenzene; PCP: pentachlorophenol; *p*,*p*'-DDE: *p*,*p*'-dichlorodiphenylene; PCB: polychlorinated biphenyl; *β*-HCH: *β*-hexachlorocyclohexane; Flu: fluorene; Ant: anthracene.

### 2.3 血清中化学污染物浓度的影响因素分析

将血清化学污染物浓度作为因变量,人口学资料、生活习惯和膳食资料作为自变量建立多元线性回归方程,分析影响血清中化学污染物浓度的因素(见表2)。结果显示,女性血清中*β*-六六六浓度高于男性,这可能是由于OCPs具有亲脂性,主要聚集在脂肪组织中,而女性体脂水平普遍高于同龄男性,因此在女性体内的残留水平更高^[29,30]^;年龄与*p*,*p*'-滴滴伊(*β*=0.032, 95% CI: 0.001~0.063)、PCB-138(*β*=0.023, 95% CI: 0.001~0.045)、PCB-153(*β*=0.039, 95% CI: 0.015~0.064)和*β*-六六六(*β*=0.043, 95% CI: 0.002~0.083)均呈正相关,与既往研究结果一致^[31,32]^,这可能是由于OCPs和PCBs具有持久性,且中老年人暴露时间长,代谢能力差,从而导致化学污染物在体内长期蓄积^[33]^;肉类摄入频率与*p*,*p*'-滴滴伊(*β*=0.071, 95% CI: 0.006~0.136)和*β*-六六六(*β*=0.102, 95% CI: 0.019~0.186)呈正相关,蔬菜摄入频率高的人群具有较高的血清五氯苯酚暴露水平(*β*=0.143, 95% CI: 0.054~0.232),膳食暴露是农药的主要暴露来源,因此,较高的肉类和蔬菜摄入频率可能会增加农药的暴露水平^[34]^;另外,饮酒者体内的五氯苯酚浓度高于不饮酒者(*β*=3.385, 95% CI: 0.616~6.154),提示饮酒可能增加该物质的暴露风险。本研究中未发现教育程度、吸烟和体育锻炼与化学污染物浓度之间的关联,需要开展更大样本量的研究来全面探讨化学污染物浓度的影响因素。

**表2:** 血清中化学污染物浓度影响因素的多元线性回归分析结果

Influencing factor		*β* (95%CI)							
		HCB	PCP	*p*, *p*'-DDE	PCB-138	PCB-153	*β*-HCH	Flu	Ant
Gender	Male	0	0	0	0	0	0	0	0
	Female	0.407 (-0.109, 0.924)	-0.802 (-1.632, 0.028)	0.528 (-0.107, 1.163)	0.034 (-0.407, 0.475)	-0.040 (-0.541, 0.462)	**1.794** **(0.974,** **2.613)**	0.909 (-0.879, 2.697)	-0.098 (-1.003, 0.806)
	Age (years)	0.017 (-0.009, 0.042)	-0.020 (-0.061, 0.020)	**0.032** **(0.001,** **0.063)**	**0.023** **(0.001,** **0.045)**	**0.039** **(0.015,** **0.064)**	**0.043** **(0.002,** **0.083)**	0.059 (-0.030, 0.147)	-0.048 (-0.092, 0.001)
	BMI (kg/m^2^)	0.030 (-0.043, 0.104)	-0.012 (-0.131, -0.106)	0.033 (-0.058, 0.124)	0.014 (-0.049, 0.077)	0.002 (-0.070, 0.074)	0.024 (-0.093, 0.141)	0.095 (-0.160, 0.350)	0.053 (-0.076, 0.182)
Educational	Middle school or below	0	0	0	0	0	0	0	0
level	High school	0.549 (-0.001, 1.099)	-0.841 (-1.724, 0.042)	0.531 (-0.144, 1.207)	0.065 (-0.405, 0.534)	0.245 (-0.289, 0.779)	0.852 (-0.020, 1.724)	0.799 (-1.104, 2.703)	-0.602 (-1.565, 0.360)
	College or above	0.192 (-0.388, 0.773)	0.064 (-0.869, 0.997)	0.463 (-0.251, 1.177)	0.317 (-0.179, 0.813)	0.519 (-0.045, 1.083)	0.377 (-0.545, 1.298)	0.854 (-1.158, 2.865)	-0.344 (-1.361, 0.673)
Smoking	Non-smoker	0	0	0	0	0	0	0	0
habit	Current smoker	-0.490 (-1.042, 0.062)	-0.416 (-1.304, 0.471)	-0.340 (-1.019, 0.339)	-0.156 (-0.628, 0.316)	-0.532 (-1.068, 0.004)	-0.411 (-1.287, 0.466)	0.743 (-1.169, 2.656)	0.532 (-0.435, 1.500)
	Former smoker	-0.529 (-1.660, 0.602)	0.742 (-1.076, 2.560)	-0.253 (-1.643, 1.138)	0.122 (-0.844, 1.088)	-0.175 (-1.273, 0.924)	0.029 (-1.766, 1.824)	-2.074 (-5.992, 1.844)	1.716 (-0.265, 3.698)
Alcohol	Non-consumer	0	0	0	0	0	0	0	0
consumption	Current comsumer	0.287 (-0.258, 0.832)	**3.385** **(0.616,** **6.154)**	0.321 (-0.350, 0.991)	0.444 (-0.022, 0.910)	0.539 (0.009, 1.069)	-0.009 (-0.874, 0.857)	0.224 (-1.666, 2.114)	-0.132 (-1.088, 0.824)
	Former comsumer	1.047 (-0.675, 2.770)	0.513 (-0.364, 1.390)	0.628 (-1.491, 2.746)	-0.034 (-1.505, 1.437)	0.807 (-0.867, 2.480)	1.761 (-0.974, 4.495)	2.183 (-3.785, 8.151)	-0.776 (-3.795, 2.242)
Physical	No	0	0	0	0	0	0	0	0
activity	Yes	-0.232 (-0.680, 0.217)	-0.175 (-0.896, 0.545)	-0.017 (-0.568, 0.534)	0.027 (-0.356, 0.410)	0.005 (-0.430, 0.441)	0.278 (-0.434, 0.989)	-0.015 (-1.568, 1.538)	-0.245 (-1.030, 0.541)
Diet	Meat (times/week)	0.051 (-0.001, 0.104)	0.003 (-0.082, 0.088)	**0.071** **(0.006,** **0.136)**	0.008 (-0.038, 0.053)	0.030 (-0.021, 0.082)	**0.102** **(0.019,** **0.186)**	0.106 (-0.077, 0.289)	-0.039 (-0.132, 0.054)
	Vegetable (times/week)	-0.006 (-0.061, 0.050)	**0.143** **(0.054,** **0.232)**	0.031 (-0.037, 0.099)	0.006 (-0.041, 0.053)	0.006 (-0.048, 0.060)	0.028 (-0.059, 0.116)	-0.057 (-0.248, 0.135)	-0.006 (-0.103, 0.091)
	Egg (times/week)	0.037 (-0.035, 0.108)	0.014 (-0.101, 0.129)	-0.020 (-0.108, 0.068)	-0.001 (-0.061, 0.061)	-0.007 (-0.077, 0.063)	-0.010 (-0.123, 0.104)	0.068 (-0.180, 0.316)	-0.065 (-0.190, 0.061)
	Fruit (times/week)	-0.026 (-0.095, 0.043)	-0.002 (-0.113, 0.108)	-0.046 (-0.130, 0.039)	-0.018 (-0.077, 0.040)	-0.013 (-0.080, 0.054)	-0.034 (-0.144, 0.075)	0.037 (-0.201, 0.276)	-0.010 (-0.130, 0.111)
	Milk (times/week)	-0.096 (-0.197, 0.005)	0.046 (-0.117, 0.208)	-0.013 (-0.137, 0.111)	-0.048 (-0.135, 0.038)	-0.078 (-0.177, 0.020)	-0.045 (-0.206, 0.115)	-0.175 (-0.525, 0.175)	0.002 (-0.175, 0.179)
	Nut (times/week)	-0.026 (-0.120, 0.067)	0.038 (-0.112, 0.189)	-0.084 (-0.199, 0.031)	-0.026 (-0.106, 0.054)	-0.009 (-0.100, 0.082)	-0.051 (-0.200, 0.098)	-0.037 (-0.362, 0.287)	0.013 (-0.151, 0.177)

Values in bold indicate statistical significance (*P*<0.05).

### 2.4 健康风险评估

健康风险评估可以反映化学污染物的暴露是否会对人群的健康状况造成不利影响,从而使生物监测数据能够在公共卫生背景下进行解释。在本研究中,BE和HBM值仅适用于五氯苯酚、六氯苯和*p*,*p*'-滴滴伊。五氯苯酚的HBM-Ⅰ(40 μg/L)和HBM-Ⅱ(70 μg/L)值是基于非致癌效应得出的^[16]^;六氯苯和*p*,*p*'-滴滴伊的BE值分别为340 ng/g脂质^[35]^和5000 ng/g脂质^[36]^。血清中五氯苯酚、六氯苯和*p*,*p*'-滴滴伊的HQ如图1所示。与HBM-Ⅰ相比,有约6.7%的研究对象血清中五氯苯酚的HQ超过1,表明人群可能存在与五氯苯酚暴露相关的健康风险;五氯苯酚属于持久性有机污染物,具有持久性、生物蓄积性和致癌性等^[37,38]^,在我国主要用于杀灭血吸虫的中间宿主钉螺,本研究所处省份属血吸虫病流行省,曾大量使用五氯苯酚及其钠盐,造成环境污染并对人体健康产生严重威胁^[39]^。在研究人群中没有观察到六氯苯和*p*,*p*'-滴滴伊的健康风险。研究人群HI的第95百分位数为2.48,约28.3%的研究对象HI值大于1,表明污染物复合暴露的健康风险不容忽视。后续应进行更全面的暴露监测和健康风险评估。

**图1:**
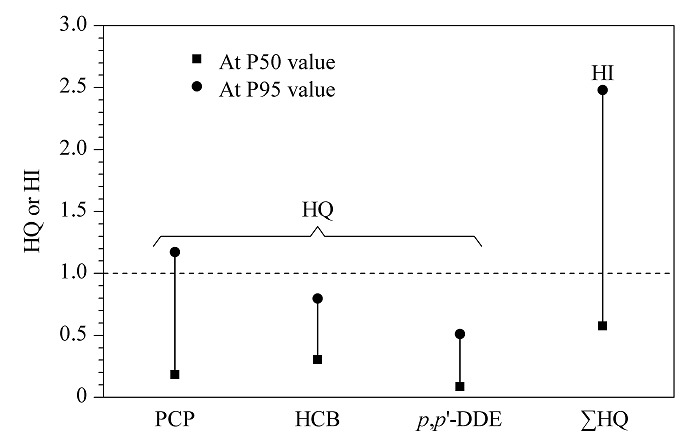
化学污染物的危害商和危害指数

## 3 结论

本研究发现我国中部地区普通成人广泛暴露于各种化学污染物,以OCPs、PCBs和PAHs为主要污染物,其中五氯苯酚是浓度水平最高的化合物。年龄、性别和饮食是血清中化学污染物浓度的重要影响因素。本研究评估了人体内化学污染物暴露水平和特征,并进行了单一和累积暴露的健康风险评估,部分人群暴露于五氯苯酚可能存在潜在的健康风险,且多污染物累积暴露风险不容忽视,未来应开展大规模的人体生物监测进一步探讨化学污染物暴露对人体健康的影响。

## References

[1] Zhu M, Yuan Y, Yin H, et al. Sci Total Environ, 2022, 805: 150270 34536863 10.1016/j.scitotenv.2021.150270

[2] Li C, Zhu H, Li C, et al. Food Chem, 2021, 354: 129552 33756332 10.1016/j.foodchem.2021.129552

[3] Seo S H, Choi S D, Batterman S, et al. J Hazard Mater, 2022, 424: 127381 34638073 10.1016/j.jhazmat.2021.127381

[4] Montalbano A M, Albano G D, Anzalone G, et al. Chemosphere, 2020, 245: 125600 31864052 10.1016/j.chemosphere.2019.125600

[5] Sobus J R, DeWoskin R S, Tan Y-M, et al. Environ Health Perspect, 2015, 123: 919 25859901 10.1289/ehp.1409177 PMC4590763

[6] Haines D A, Saravanabhavan G, Werry K, et al. Int J Hyg Environ Health, 2017, 220: 13 27601095 10.1016/j.ijheh.2016.08.002

[7] Apel P, Angerer J, Wilhelm M, et al. Int J Hyg Environ Health, 2017, 220: 152 27914867 10.1016/j.ijheh.2016.09.007

[8] Cao Z, Lin S, Zhao F, et al. Environ Int, 2021, 146: 106252 33242729 10.1016/j.envint.2020.106252 PMC7828642

[9] Sun D, Song Z, Zhang Y, et al. Front Environ Chem, 2021, 2: 703961

[10] Qu G, Chen B, Liu S, et al. J Anal Test, 2023, 7: 163

[11] Fu L, Song S, Luo X, et al. Environ Pollut, 2023, 337: 122580 37734633 10.1016/j.envpol.2023.122580

[12] Shang N, Yang Y, Xiao Y, et al. Environ Pollut, 2023, 342: 123069 38052341 10.1016/j.envpol.2023.123069

[13] Li X. [MS Dissertation]. Wuhan: Huazhong University of Science and Technology, 2022

[14] Covaci A, Voorspoels S, Thomsen C, et al. Sci Total Environ, 2006, 366: 361 16624383 10.1016/j.scitotenv.2006.03.006

[15] Hays S M, Aylward L L, LaKind J S, et al. Regul Toxicol Pharmacol, 2008, 51: S4 18423822 10.1016/j.yrtph.2008.02.007

[16] Schulz C, Angerer J, Ewers U, et al. Int J Hyg Environ Health, 2007, 210: 373 17337242 10.1016/j.ijheh.2007.01.035

[17] Aylward L L, Kirman C R, Schoeny R, et al. Environ Health Perspect, 2013, 121: 287 23232556 10.1289/ehp.1205740 PMC3621178

[18] Deering K, Spiegel E, Quaisser C, et al. Environ Res, 2020, 184: 109271 32143026 10.1016/j.envres.2020.109271

[19] Dufour P, Pirard C, Charlier C. Sci Total Environ, 2017, 599/600: 1856 28545212 10.1016/j.scitotenv.2017.05.157

[20] Helou K, Harmouche-Karaki M, Karake S, et al. Chemosphere, 2019, 231: 357 31136903 10.1016/j.chemosphere.2019.05.109

[21] Kim M J, Choi S, Kim S. Environ Res, 2022, 212: 1131434

[22] Sun J, Pan L, Tsang D C W, et al. Sci Total Environ, 2018, 615: 724 29017123 10.1016/j.scitotenv.2017.09.271

[23] Yu H, Liu Y, Shu X, et al. Chemosphere, 2020, 243: 125392 31995868 10.1016/j.chemosphere.2019.125392

[24] Deziel N C, Warren J L, Huang H, et al. Environ Res, 2021, 192: 110333 33068584 10.1016/j.envres.2020.110333 PMC7736223

[25] Haines D A, Saravanabhavan G, Werry K, et al. Int J Hyg Environ Health, 2017, 220: 13 27601095 10.1016/j.ijheh.2016.08.002

[26] Xu P, Lou X, Ding G, et al. Sci Total Environ, 2015, 536: 215 26218560 10.1016/j.scitotenv.2015.07.025

[27] Xia X, Carroll-Haddad A, Brown N, et al. J Occup Environ Med, 2016, 58: S72 27501107 10.1097/JOM.0000000000000743 PMC4977992

[28] Wang T, Li B J, Liao H, et al. Sci Total Environ, 2021, 789: 148003 34323836 10.1016/j.scitotenv.2021.148003

[29] Freire C, Koifman R J, Koifman S. Sci Total Environ, 2017, 598: 722 28456124 10.1016/j.scitotenv.2017.04.128

[30] Chang C, Chen M, Gao J, et al. Environ Int, 2017, 102: 213 28284820 10.1016/j.envint.2017.03.004

[31] Han M, Ma A, Dong Z, et al. Sci Total Environ, 2023, 860: 160358 36436633 10.1016/j.scitotenv.2022.160358

[32] Li X, Wang L M, Song L L, et al. Chinese Journal of Chromatography, 2022, 40(5): 461 35478005 10.3724/SP.J.1123.2021.12013 PMC9404153

[33] Li J, Wang P, Shi S, et al. Environ Monit Assess, 2018, 190: 315 29705822 10.1007/s10661-018-6694-3

[34] Van Audenhaege M, Heraud F, Menard C, et al. Food Addit Contam Part A Chem Anal Control Expo Risk Assess, 2009, 26: 1372 19707917 10.1080/02652030903031171

[35] Aylward L L, Hays S M, Gagné M, et al. Regul Toxicol Pharmacol, 2010, 58: 25 20547196 10.1016/j.yrtph.2010.06.003

[36] Kirman C R, Aylward L L, Hays S M, et al. Regul Toxicol Pharmacol, 2011, 60: 172 21466830 10.1016/j.yrtph.2011.03.012

[37] Huo Y, Wan Y, Huang Q, et al. Sci Total Environ, 2022, 831: 154889 35364152 10.1016/j.scitotenv.2022.154889

[38] Sun Y, Liu Z, Xia W, et al. Environ Sci Pollut Res Int, 2023, 30: 37598 36574129 10.1007/s11356-022-24802-y

[39] Zhang L J, He J Y, Yang F, et al. Chinese Journal of Schistosomiasis Control, 2023, 35(3): 217 37455091 10.16250/j.32.1374.2023073

